# Freestanding Needle Flower Structure CuCo_2_S_4_ on Carbon Cloth for Flexible High Energy Supercapacitors With the Gel Electrolyte

**DOI:** 10.3389/fchem.2020.00062

**Published:** 2020-02-27

**Authors:** Tian Xie, Jinxiao Xu, Jie Wang, Chuanli Ma, Linghao Su, Fengying Dong, Liangyu Gong

**Affiliations:** College of Chemistry and Pharmaceutical Sciences, Qingdao Agricultural University, Qingdao, China

**Keywords:** flexible electrode, nanoneedles, CuCo_2_S_4_, pseudocapacitance, supercapacitors

## Abstract

A facile hydrothermal approach was adopted to the direct synthesis of bimetallic sulfide (CuCo_2_S_4_) on carbon cloth (CC) without binders for the supercapacitor's electrodes. A possible formation mechanism was proposed. The prepared bimetallic electrode exhibited a high specific capacitance (Csp) of 1,312 F·g^−1^ at 1 A·g^−1^, and an excellent capacitance retention of 94% at 5 A·g^−1^ over 5,000 cycles. In addition, the asymmetric supercapacitor (CuCo_2_S_4_/CC//AC/CC) exhibited energy density (42.9 wh·kg^−1^ at 0.8 kW·kg^−1^) and outstanding cycle performance (80% initial capacity retention after 5,000 cycles at 10 A·g^−1^). It should be noted that the electrochemical performance of a supercapacitor device is quite stable at different bending angles. Two charged devices in series can light 28 red-colored LEDs (2.0 V) for 5 min. All of this serves to indicate the potentially high application value of CuCo_2_S_4_.

## Introduction

Supercapacitors are extremely valuable for energy storage, owing to their rapid rechargeable ability and extended life cycle (Gao et al., 2015b; He and Chen, 2015). In recent years, they have been widely applied in many fields, such as power backup devices, hybrid electric vehicles, and portable and wearable electronic products (Dong et al., 2015). While flexible electronic products have experienced explosive growth, all electronic devices require flexible and long-life energy storage systems (Hu et al., 2010; Shen et al., 2014; Liu G. et al., 2018). Currently, the main commercial supercapacitors use carbon materials as electrode materials and thus suffer from low-energy density (Dong et al., 2016). Therefore, designing advanced nanostructures with high-energy density and outstanding performance at high-current densities is still given a large amount of attention and holds great appeal (Bao and Li, 2012; Wang et al., 2014; Huang et al., 2016; Xing et al., 2018).

Transition metal compounds, including oxides and sulfides, can provide superior specific capacitance (C_sp_) due to the rapid faraday redox reaction (Guo et al., 2014; Padmanathan and Selladurai, 2014; Pu et al., 2014; Gao et al., 2015b; Moosavifard et al., 2016). However, the maximum capacitance and energy density of these materials are severely limited by the lower electrical conductivity. Among them, transitional bimetallic sulfides can provide a richer redox reaction, resulting in a generally superior electrochemical performance (Xiong et al., 2015; Sun et al., 2017; Wang J. et al., 2018; You et al., 2018; Dong et al., 2019). For example, Li et al. supported NiCo_2_S_4_ nanorods on Ni foam, which displayed a 94.96% capacitive retention over 10,000 cycles (Li M. L. et al., 2018; Li X.-X et al., 2018). At the same time, it has been found that transition bimetallic sulfides also possess rich redox couples and enhance the electronic conductivity (Ai et al., 2016; Wang et al., 2017). In recent years, CuCo_2_S_4_ as electrode material has been found to have excellent electronic conductivity, good electrochemical stability, and higher conductivity when compared to its oxide counterparts. In addition, the synergistic effects due to the interaction between diverse metal compounds leads to an enhanced electrochemical energy storage performance, as compared with monometallic sulfides (Shinde et al., 2019). For example, Tian et al. anchored CuCo_2_S_4_ to graphene aerogel through solvent thermal reaction with 668 F·g^−1^ at 1A·g^−1^. Asymmetrical supercapacitors based on CuCo_2_S_4_/GA exhibit a maximum of 22 Wh·kg^−1^ (Tian et al., 2018). Guo et al. prepared Co_2_CuS_4_ on graphene nanosheets, which exhibited C_sp_ of 1,005 F·g^−1^ at 1A·g^−1^ (Guo et al., 2016). In addition, designing nanostructures with large surface areas is important for promoting ion transport in electrochemical processes (Wang et al., 2016; Shang et al., 2018).

In order to adapt to the trend of wearable and flexible supercapacitors, others found it desirable to prepare electrode materials on flexible substrates (Dingshan et al., 2015; He et al., 2018). According to previous reports, carbon fiber, nickel foam, and copper foam are widely applied as conductive substrate (Martti et al., 2009; Yang et al., 2014; Xiao et al., 2015; Sun et al., 2016). Among them, the carbon fiber cloth is considered to be an ideal conductive substrate due to its flexibility, electrical conductivity, and low cost. At the same time, directly growing nanostructured materials on carbon cloth avoids the use of binders, resulting in rapid electro transport (Jiang et al., 2011; Liu et al., 2011; Yuan et al., 2012; Zhang et al., 2012, 2016; Wang et al., 2013).

Here, we confirm that carbon cloth is a conductive substrate that can be used to grow a novel 3D needle-flowerlike CuCo_2_S_4_ nanostructure. As a result, the flowerlike CuCo_2_S_4_ nanostructure exhibits excellent performance (1,312 F·g^−1^ at 1 A·g^−1^). Beyond that, an asymmetric solid-state device was manufactured. As a result, it's provided a significant energy density of 42.9 Wh·kg^−1^ with an initial capacitance retention rate of 80%–even after 5,000 cycles in the 1.6 V-wide working potential window, showing excellent cycling stability.

## Materials and Methods

### Preparation of CuCo_2_S_4_/CC

All reagents were analytical-grade and used without any additional purification. The carbon cloth was purchased from Ce Tech WOS1009. First, a piece of carbon cloth was refluxed at 100°C for 1 h with HNO_3_ and subsequently rinsed with deionized water and ethanol. As is typical, CuCl_2_·6H_2_O (2 mmol), CoCl_2_·2H_2_O (4 mmol), urea (30 mmol), and NH_4_F (20 mmol) were dissolved into 60 mL DI water and stirred for 10 min. Then, the mixture solution and the treated CC were transferred into 100 mL autoclave. After the hydrothermal reaction (140°C for 6 h), the precursors on carbon cloth were washed with deionized water and ethanol, and then dried at 60°C for 12 h.

Next, thioacetamide (TAA, 5 mmol) was dissolved in 60 mL of absolute ethanol with stirring. Then, the solution and the as-obtained precursor were transferred together to the 100 ml autoclave (140°C for 6 h). Finally, after being washed and dried at 60°C overnight, the carbon cloth of uniform load CuCo_2_S_4_ was obtained. The loading mass of CuCo_2_S_4_ was about 2.3 mg·cm^−2^. For comparison, CuCo_2_S_4_ was directly obtained without the placement of a carbon cloth and the CC without the loaded sample is named blank-CC.

### Characterization

X-ray diffraction (XRD) patterns were recorded on a Bruker D8. The morphology of samples was studied by scanning electron microscopy (SEM) (JEOL-7500F, Japan) and transmission electron microscopy (TEM) (HITACHI-7650, Japan). Cycling voltammetry (CV), galvanostatic charge-discharge (GCD), and electrochemical impedance spectroscopy (EIS) were performed with a CHI760E (Shanghai CH, China) electrochemical workstation. Additionally, the cycle life was tested on a CT2001A LAND Battery Test System (LAND, Wuhan, China).

### Electrochemical Measurements

The electrochemical measurements were carried out in a three-electrode system by using 3 M KOH aqueous solution as the electrolyte, where the CuCo_2_S_4_/CC was directly used as the working electrode, Pt foil was used as the counter electrode, and a saturated calomel electrode (SCE.) was used as the reference electrode. The C_sp_ of materials was calculated through the equation *C*_*sp*_ = *I* × Δ*t* / *m* × Δ*V*, where I, ρt, m, and V are the discharge current (A), discharge time (s), loading mass (g), and voltage range.

The flexible solid-state asymmetric supercapacitor (FSASC) device was assembled with a CuCo_2_S_4_/CC cathode, active carbon (AC)/CC anode, and PVA/KOH gel electrolyte. The mass ratio between CuCo_2_S_4_/CC and AC/CC was m^+^/m^−^ = 0.19, according to charge conservation (Yuan et al., 2018). The AC/CC electrode was produced by mixing AC 80 wt%, acetylene black 10 wt%, and PTFE 10 wt% and then coated onto carbon cloth. The PVA/KOH gel electrolyte was maintained by mixing KOH (3 g) and PVA (3 g) in 30 ml DI water at 80°C for 1 h with stirring. The energy and power densities were measured by using equation *E (*Wh·k*g*^−1^*)* = *C*_*sp*_ × Δ*V*^2^ / 2 and *P (W*·*kg*^−1^*)* = *E /* Δ*t*.

## Results and Discussion

**Scheme 1:**
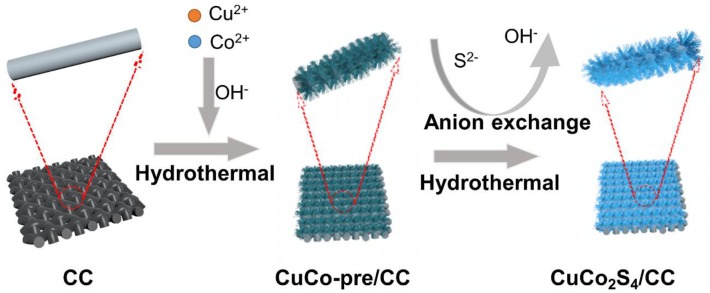
The growth mechanism schematic of CuCo_2_S_4_ on carbon cloth.

As illustrated in Scheme 1, the CuCo_2_S_4_/CC was successfully synthesized through a simple two-step process. First, the Cu-Co precursor was directly grown on the CC via a hydrothermal process. Subsequently, the Cu-Co precursor is converted into CuCo_2_S_4_/CC via a sulfurization strategy.

**Figure 1:**
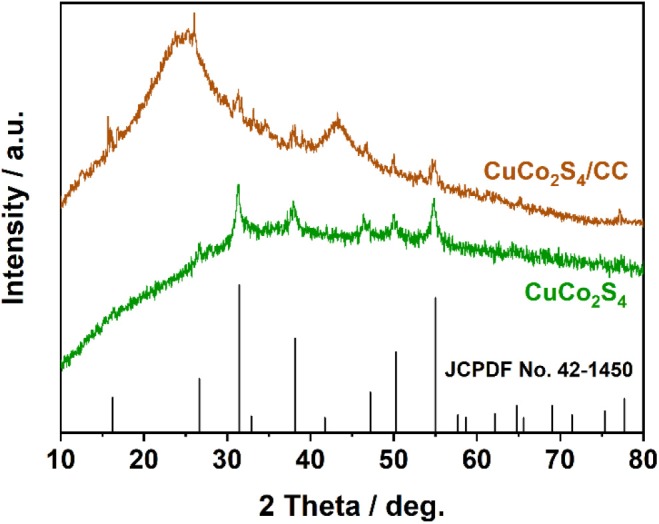
XRD patterns of CuCo_2_S_4_/CC and CuCo_2_S_4_.

The chemical composition is recorded by XRD (Figure 1). The different peaks of 16, 26, 31.2, 37.9, 46.9, 49.9, and 54.8° are identical to the standard diffraction data of CuCo_2_S_4_ (JCPDS No.42-1450) (You et al., 2018). And the broad peaks at 25 and 45° are derived from the CC substrate (Liu and Wu, 2018).

**Figure 2:**
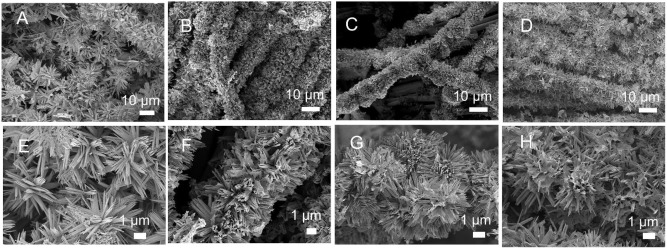
SEM images for CuCo_2_S_4_/CC at different reaction times: 2 h **(A,E)**; 4 h **(B,F)**; 6 h **(C,G)**, and 8 h **(D,H)**.

Figures 2A–H shows the topographical changes of CuCo_2_S_4_ on carbon cloth. In Figure 2A, the CuCo_2_S_4_ was uniformly adhered to the carbon fiber at a reaction time of 2 h. As seen in Figure 2E, the flowerlike structure, composed of acicular CuCo_2_S_4_, is supported on the carbon fiber. When the reaction time was extended to 4 h (Figures 2B,F), the acicular structure of CuCo_2_S_4_ began to aggregate. When the reaction time was 6 h (Figures 2C,G), the acicular structure was further aggregated to form a flowerlike structure. The acicular structure provides more active sites, providing abundant pores, while the surface that is initially aggregated by the needle structure provides a larger reaction area. When the reaction time was extended to 8 h, the flowerlike structure supported on the carbon fiber is composed of agglomerated sheets, which reduces the reactive sites and electrochemical performance (Figures 2D,H). The morphological structure of the CoCu-pre and CuCo_2_S_4_ is shown in Figure S1. In Figure S1A, the needle CuCo-pre constitutes a flowerlike structure and uniformly grows on the carbon fibers. Figure S1B clearly shows the flowerlike CuCo_2_S_4_ structure on the carbon fiber; the exchange of anions during vulcanization does not destroy the overall morphology. In contrast, the CuCo-pre and CuCo_2_S_4_ were obtained without adding a carbon cloth substrate (Figures S1C,D). It can be seen that both exhibit similar morphologies as those loaded on carbon cloth.

**Figure 3:**
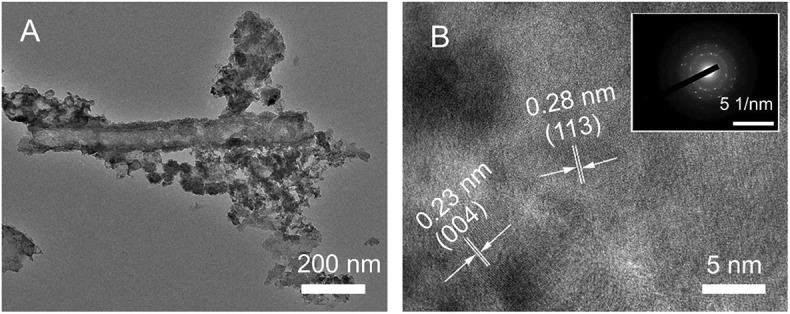
TEM image **(A)**; HRTEM image **(B)**; and SAED pattern (inset) of CuCo_2_S_4_/CC.

In addition, the microstructure of the CuCo_2_S_4_/CC shown by TEM (Figure 3A) further demonstrates that the acicular structure of CuCo_2_S_4_ is attached to the carbon fibers. A HRTEM image shows clear lattice fringes with lattice spacing corresponding to the (113) and (004) planes (Figure 3B). The SAED in inset indicates the polycrystalline of the materials. Meanwhile, the TEM and SAED image of CuCo_2_S_4_ shows that it is composed of many primary nano-needles and its polycrystalline (Figures S1E,F).

Through the above observations, the growth mechanism of flowerlike CuCo_2_S_4_ on carbon cloth can be preliminarily obtained. First, in an alkaline environment, Cu^2+^ and Co^2+^ ions form a CuCo(OH)_x_ precursor (Wan et al., 2015; Jayaraman et al., 2017). At this time, the solution becomes a supersaturated solution with a large amount of Cu-Co precursor particles. Due to the high surface energy and thermodynamic instability, the nanoparticles adhere to the carbon fibers to reduce the surface energy. Based on the hydrolysis rate control of NH_4_F, the precursors will be oriented to grow into the acicular structure and further assemble into a flowerlike structure (Liu S. et al., 2018). The second step is the vulcanization process. The S^2−^ released by TAA undergoes anion exchange with OH^−^ to form a CuCo_2_S_4_ flowerlike structure.

**Figure 4:**
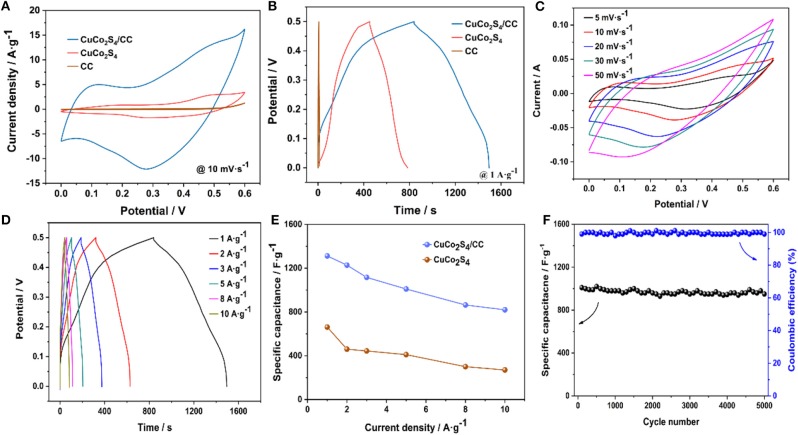
CV curves of CuCo_2_S_4_/CC, CuCo_2_S_4_, and blank-CC at 10 mV·s^−1^ **(A)**; GCD curves of CuCo_2_S_4_/CC, CuCo_2_S_4_, and blank-CC at 1A·g^−1^ **(B)**; CV curves of CuCo_2_S_4_/CC at 5–50 mV·s^−1^ **(C)**; GCD curves of CuCo_2_S_4_/CC at 1–10A·g^−1^ **(D)**; the rate capability of the as-prepared two electrodes **(E)**; Cycling performance at 5A·g^−1^ **(F)**.

The electrochemical properties of CuCo_2_S_4_/CC were measured. Figure 4A shows the CV curve of CuCo_2_S_4_/CC, CuCo_2_S_4_ and blank-CC at 10 mV·s^−1^. The curve area of CuCo_2_S_4_/CC is larger than that of CuCo_2_S_4_ and blank-CC, which means that CuCo_2_S_4_/CC has a higher specific capacitance. Figure 4B is a CuCo_2_S_4_/CC and CuCo_2_S_4_ GCD curves at 1 A·g^−1^. Similarly, the discharge time of the CuCo_2_S_4_/CC material is much higher than that of CuCo_2_S_4_. And, the capacitance contribution of blank-CC is negligible. Figure 4C shows typical CV curves for CuCo_2_S_4_/CC at 0–0.6 V. Each curve shows a significant redox peak, indicating that the Cu(Co)-S-O/Cu(Co)-S-OH-related faraday reaction was carried out (Jayaraman et al., 2017). As the scan rate increased, the redox peak appeared to shift due to the presence of polarization (Ratha et al., 2017). The possible electrochemical reaction equations are as follows (Kaverlavani et al., 2017; Zhu et al., 2017):

(1) $$\begin{array}{r} \text{CuC}{\text{o}}_{2}{\text{S}}_{4}+\text{O}{\text{H}}^{-}+{\text{H}}_{2}\text{O}↔\text{CuSOH}+2\text{CoSOH}+{\text{e}}^{-} \end{array}$$

(2) $$\begin{array}{r} \text{CuSOH}+\text{O}{\text{H}}^{-}↔\text{CuSO}+{\text{H}}_{2}\text{O}+{\text{e}}^{-} \end{array}$$

(3) $$\begin{array}{r} \text{CoSOH}+\text{O}{\text{H}}^{-}↔\text{CoSO}+\text{H}2\text{O}+{\text{e}}^{-} \end{array}$$

In order to accurately compare the specific capacitances, the exact C_sp_ was calculated from GCD measures. Figure 4D shows the GCD curves of CuCo_2_S_4_/CC from 1 to 10 A·g^−1^. From the comparison of C_sp_ graphs of CuCo_2_S_4_/CC and CuCo_2_S_4_ (Figure 4E), the discharge-specific capacitance of CuCo_2_S_4_/CC is 1,312, 1,228, 1,116, 1,010, 864, and 820 F·g^−1^ at 1, 2, 3, 5, 8, 10 A·g^−1^, which is much larger than CuCo_2_S_4_. The specific capacity values were compatible relative to those previously reported (Table S1). On the other hand, lower charge transfer resistance (R_ct_) and Warburg impedance (W) also means that CuCo_2_S_4_/CC can show superior electrochemical performance compared to CuCo_2_S_4_ (Figure S2). It is noteworthy that the capacities decrease with an increase in current density, which may be due to the fact that the transport and/or diffusion of electrolyte ions are suppressed in high current density (Brousse et al., 2015). The CuCo_2_S_4_/CC electrode maintained an initial capacity of 94% after cycling 5,000 times at 5 A·g^−1^, while the Coulomb efficiency remained almost 100%, indicating excellent cycling stability (Figure 4F).

**Figure 5:**
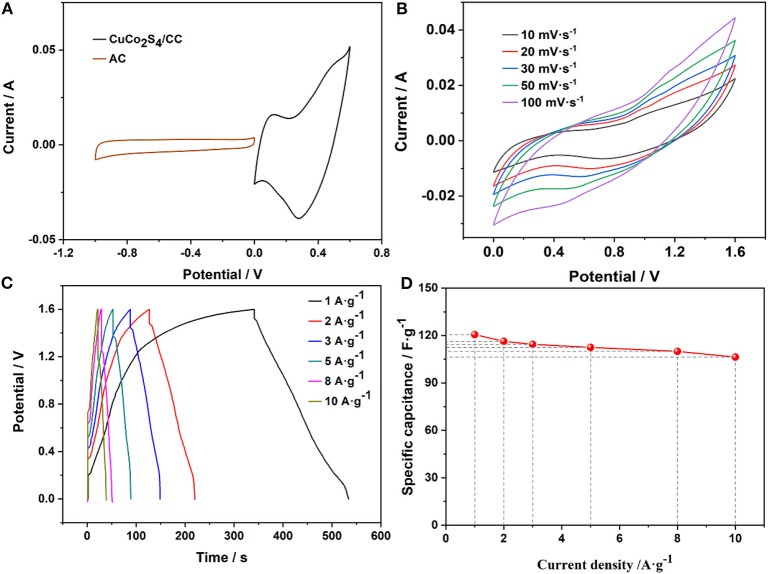
CV curves of the positive and negative **(A)**; CV curves **(B)**; GCD curves **(C)**; and the rate characteristics **(D)** of the device.

To prove the application potential, the FSASC device was made of CuCo_2_S_4_/CC and AC/CC as the positive and negative electrodes, respectively. Figure 5A is the CV curves of CuCo_2_S_4_/CC and AC/CC at 20 mV·s^−1^. The detailed electrochemical data of the AC is shown in Figure S3. The electrochemical properties of the assembled FSASC were evaluated. The CV and GCD measurements of the ASC device are shown in Figures 5B,C, respectively. At the same time, the charging time of the device is higher than the discharge time in GCD curves, which may be due to the fact that transport of the electrolyte ion cannot be fully carried out at a low current density (Figure 5C). This phenomenon stems from the nature of the gel electrolyte and the inherent properties of the material (Qi et al., 2015; Wang P. et al., 2018). When the current density is 10 A·g^−1^, a high C_sp_ of 106.3 F·g^−1^ can be delivered, which shows a retention of 88.1% (Figure 5D).

**Figure 6:**
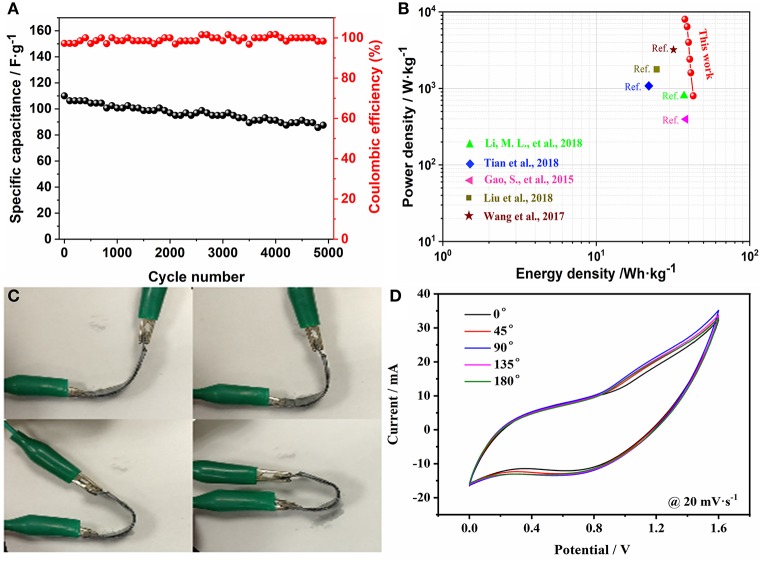
Cycling properties of the device at 10 A·g^−1^ **(A)**; Ragone plot **(B)**; images of asymmetric device at different bending states **(C)**; and the corresponding CV curves **(D)**.

The cycle properties of the ASC device were evaluated by cycling 5,000 times at 10 A·g^−1^ (Figure 6A). The C_sp_ decreases from 110 to 87.5 F·g^−1^, which exhibits an excellent retention of 80%. Meanwhile, the coulombic efficiency is almost maintained at 100%, which indicates that the prepared device has excellent performance. Figure 6B shows a maximum energy density of 42.9 Wh·kg^−1^ at 0.8 kW·kg^−1^. And it's still has 37.8 Wh·kg^−1^ at 8 kW·kg^−1^. In addition, the assembled FSASC device exhibits outstanding flexibility—the CV curves at different angles proving that the bending has little effect on the device (Figures 6C,D).

**Figure 7:**
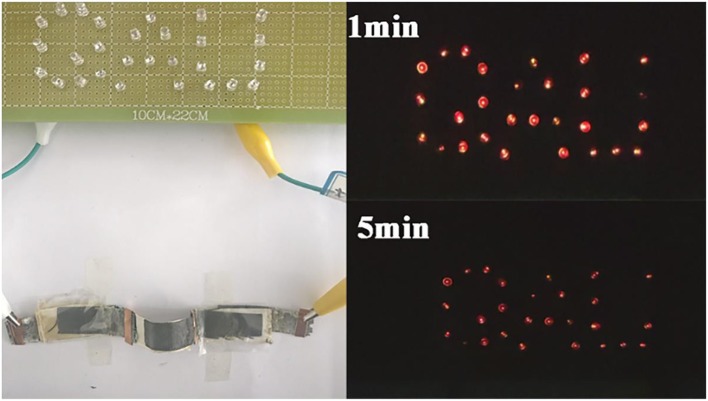
Commercial LED lights powered by three ASC devices connected in series.

To demonstrate the practical application potential of the equipment, 28 red LEDs assembled in parallel can be illuminated for more than 5 min (Figure 7), which indicates that CuCo_2_S_4_ possess excellent energy storage potential.

## Conclusions

In short, we directly grew needle flower structure CuCo_2_S_4_ on carbon cloth by means of a hydrothermal reaction and vulcanization process; the prepared CuCo_2_S_4_/CC shows an excellent electrochemical performance (1,312 F·g^−1^ at 1 A·g^−1^ and 94% retention over 5,000 cycles). When used as an electrode material for flexible asymmetric supercapacitors, the device provides higher energy density (maximum of 42.9 Wh·kg^−1^) and power density (maximum of 8 kW·kg^−1^), long-term cycling stability (80% retention over 5,000 cycles), and superior flexibility. The needle flower structure promotes the transport of electrolyte ions, providing a higher specific capacitance. The rapid transport of ions/electrons on carbon fibers imparts high-rate characteristics to flexible solid-state supercapacitors. This indicates that CuCo_2_S_4_/CC is promising as a flexible supercapacitor electrode material.

## Data Availability Statement

All datasets generated for this study are included in the article/Supplementary Material.

## Author Contributions

The manuscript was written through contributions of all authors. All authors have given their approval of the final version of the manuscript.

### Conflict of Interest

The authors declare that the research was conducted in the absence of any commercial or financial relationships that could be construed as a potential conflict of interest.
