# Supplementary material for: Freestanding Needle Flower Structure CuCo2S4 on Carbon Cloth for Flexible High Energy Supercapacitors With the Gel Electrolyte
Source: Front Chem. 2020 Feb 27;8:62. doi: 10.3389/fchem.2020.00062 (PMC7056745; doi:10.3389/fchem.2020.00062)
Supplement: Supplementary file 1 [file Data_Sheet_1.pdf]

Supplementary Material

Table S1. The comparison $CuCo_2S_4$/CC prepared in this work and other previously reported works.

Sample	Synthesis method	Specific capacitance	Cycle	Capacitance retention	Ref.
$CuCo_2S_4$/CC	Hydrothermal	456 $F{\cdot}g^{-1}$ at 1 $A{\cdot}g^{-1}$	5000	83% at 5 $A{\cdot}g^{-1}$	Yuan et al 2018
$CuCo_2O_4$/Ni	Hydrothermal/ annealing	820 $F{\cdot}g^{-1}$ at 2 $mA{\cdot}cm^{-2}$	1500	94% at 6.44 $mA{\cdot}cm^{-2}$	Wang et al 2018
$CuCo_2O_4@MnO_2$/CF	Hydrothermal	327 $F{\cdot}g^{-1}$ at 1.25 $A{\cdot}g^{-1}$	2000	93% at 1.25 $A{\cdot}g^{-1}$	Wang et al 2013
Ni/Al-LDH/CC	Hydrothermal	359 $F{\cdot}g^{-1}$ at 0.3 $A{\cdot}g^{-1}$	3000	105.9% at 1 $A{\cdot}g^{-1}$	Li et al 2017
CNT/Fe_2O_3/CC	CVD	787.5 $F{\cdot}g^{-1}$ at 5 $mV{\cdot}s^{-1}$	7000	92% at 10 $A{\cdot}g^{-1}$	Zhang et al 2017
$NiCo_2S_4$/Ni	Hydrothermal	8.85 $F{\cdot}cm^{-2}$ at 10 $mA{\cdot}cm^{-2}$	10000	94.96% at 100 $mV{\cdot}s^{-1}$	Li et al 2018
Co-Ni-OH/rGO/CC	Hydrothermal	151.46 $F{\cdot}g^{-1}$ at 2.5 $A{\cdot}g^{-1}$	1000	88.8% at 10 $A{\cdot}g^{-1}$	Wang et al 2019
$CuCo_2S_4$/CC	Hydrothermal	1312 $F{\cdot}g^{-1}$ at 1 $A{\cdot}g^{-1}$	5000	94% at 5 $A{\cdot}g^{-1}$	Our work

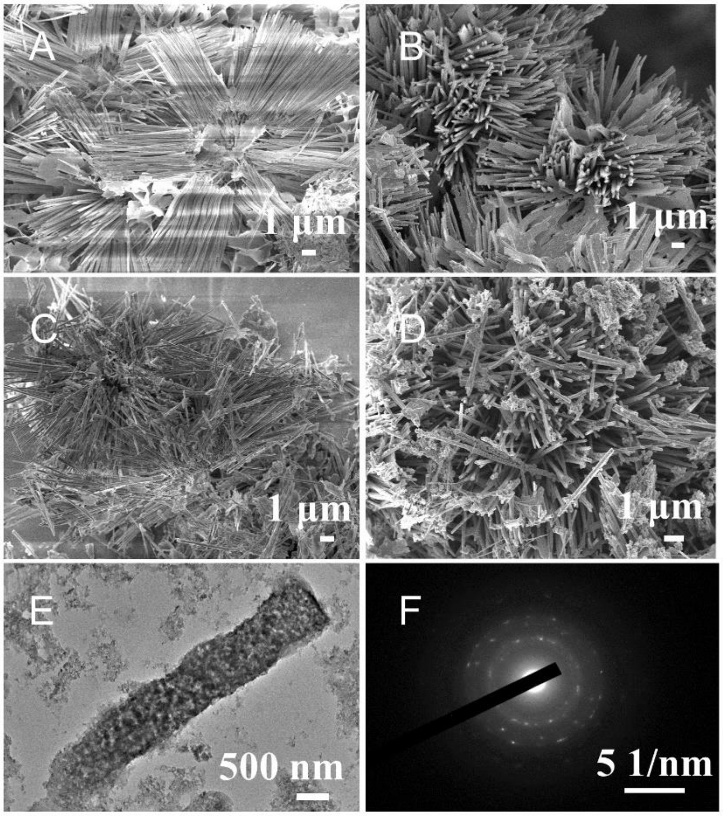

Figure S1. SEM images of samples: (A) CuCo-pre/CC, (B) $CuCo_2S_4$/CC, (C) CuCo-pre, (D) $CuCo_2S_4$; and TEM (E) and SAED (F) images of $CuCo_2S_4$.

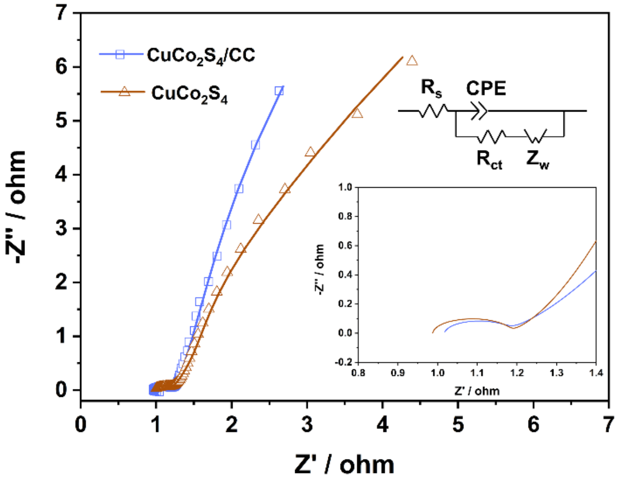

Figure S2. The EIS plots of $CuCo_2S_4$/CC and $CuCo_2S_4$.

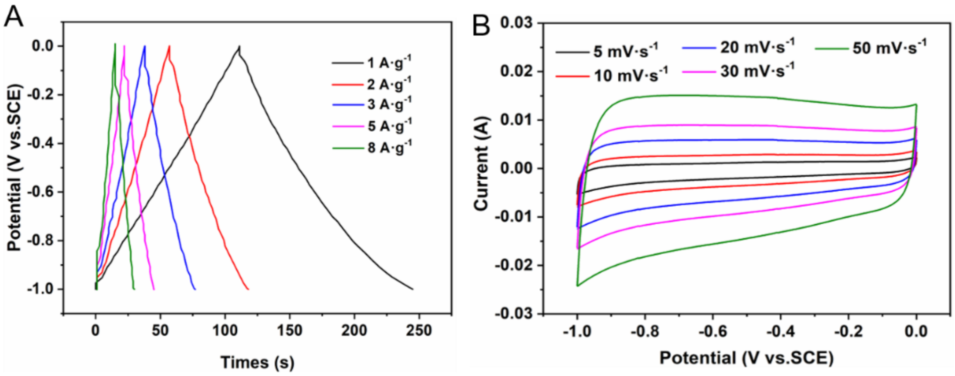

Figure S3. (A) CV curves of AC at various scan rate, (B) GCD curves of AC at various current density

References

Yuan, X., Tang, B., Sui, Y., Huang, S., Qi, J., Pu, Y., et al. (2018). $CuCo_2S_4$ nanotubes on carbon fiber papers for high-performance all-solid-state asymmetric supercapacitors. *Journal of Materials Science: Materials in Electronics* 29(10), 8636-8648. doi: 10.1007/s10854-018-8878-6.

Wang, Y., Yang, D., Lian, J., Wei, T. and Sun, Y. (2018). Ordered corn-like $CuCo_2O_4$ nanoforests covering Ni foam for a high-performance all-solid-state supercapacitor. Journal of Alloys and Compounds 741, 527-531.doi: 10.1016/j.jallcom.2018.01.168.

Wang, Q., Xu, J., Wang, X., Liu, B., Hou, X., Yu, G. et al. (2013). Core–Shell $CuCo_2O_4$@MnO_2 Nanowires on Carbon Fabrics as High-Performance Materials for Flexible, All-Solid-State, Electrochemical Capacitors. *ChemElectroChem* 1(3), 559-564. Doi: 10.1002/celc.201300084.

Li, D., Li, Y., Zhao, J., Xu, Z. and Zhang, H. (2017). Three-dimensional porous layered double hydroxides growing on carbon cloth as binder-free electrodes for supercapacitors. *Journal of Materials Research* 32(13), 2487-2496. doi: 10.1557/jmr.2017.227.

Zhang, Z., Wang, H., Zhang, Y., Mu, X., Huang, B., Du, J. et al. (2017). Carbon nanotube/hematite core/shell nanowires on carbon cloth for supercapacitor anode with ultrahigh specific capacitance and superb cycling stability. Chemical Engineering Journal 325, 221-228. doi: 10.1016/j.cej.2017.05.045.

Li, X.X., Wang, X.T., Xiao, K., Ting, O., Li, N. and Liu, Z.Q. (2018). In situ formation of consubstantial $NiCo_2S_4$nanorod arrays toward self-standing electrode for high activity supercapacitors and overall water splitting. Journal of Power Sources 402, 116-123. doi: 10.1016/j.jpowsour.2018.09.021.

Wang, D., Wei, A., Tian, L., Mensah, A., Li, D. and Xu, Y. (2019). Nickel-cobalt layered double hydroxide nanosheets with reduced graphene oxide grown on carbon cloth for symmetric supercapacitor. *Applied Surface Science* 483, 593-600. doi: 10.1016/j.apsusc.2019.03.345.
